# The Emotional Vaccine: Maternal Caregiving in Infancy Shaped Future Preschoolers' Internalizing Symptoms During the COVID‐19 Pandemic

**DOI:** 10.1111/cdev.14250

**Published:** 2025-05-30

**Authors:** Yael Schlesinger, Yael Paz, Sofie Rousseau, Naama Atzaba‐Poria, Tahl I. Frenkel

**Affiliations:** ^1^ Baruch Ivcher School of Psychology, Reichman University Herzliya Israel; ^2^ DUET Center and the Department of Psychology Ben‐Gurion University of the Negev Beer Sheva Israel; ^3^ The Paul Baerwald School of Social Work and Social Welfare The Hebrew University of Jerusalem Jerusalem Israel

**Keywords:** COVID‐19, Infancy, maternal caregiving

## Abstract

The present study assessed both concurrent and early influences of the maternal caregiving environment to examine unique contributions of each to variation in children's emotional responses to COVID‐19 pandemic. Preschoolers (3–5 years; *M* = 4.12, SD = 0.49) previously assessed in infancy, several years prior to pandemic outbreak, were re‐assessed during pandemic‐related nationwide lockdown (*N* = 200; 50% female; 63.5% secular Jews; 2016; 2021). Maternal stress during lockdown significantly moderated (*β* = 0.13, *p* < 0.05) and mediated (*β* = 0.08, *p* < 0.05) concurrent associations between preschoolers' dose of exposure (DOE) to COVID‐19 psychosocial stressors and symptoms. Furthermore, maternal sensitive care observed in infancy significantly moderated future associations between preschoolers' DOE and symptoms (*β* = −0.16, *p* < 0.05). Longitudinal protective effects of infant care remained significant after controlling for caregiver stress and behavior during the lockdown.

The COVID‐19 pandemic was a large‐scale psychosocial stressor that imposed detrimental impacts on children's mental health (Nearchou et al. 2020; Panchal et al. 2021). Recent meta‐analyses have revealed widespread emotional and behavioral impacts on children across the globe. Specifically, approximately 30%–50% of children, ranging in age from infancy to 18 years, were found to be suffering from internalizing symptoms (e.g., depression, anxiety) and externalizing symptoms (e.g., irritability and inattention) during pandemic‐related lockdowns and ongoing social isolation (Ma et al. 2021; Panda et al. 2021).

Although the pandemic represented a universal large‐scale stressor experienced worldwide, not everyone was equally exposed to its aversive features. Studies conducted during the pandemic (Brown et al. 2020a; Shorer and Leibovich 2020; Spinelli et al. 2020) revealed significant variations in levels of exposure to pandemic‐related psycho‐social stressors (termed dose of exposure/DOE), such as the intensity of negative impacts on a family's economic security, level of added parental burdens due to the pandemic, extent of exposure to COVID‐related information through the media, and extent to which close acquaintances were impacted by the pandemic (e.g., a close acquaintance was sick/lost a job, etc.). Literature on previous large‐scale stressors including natural disasters, war, and terrorism have demonstrated a dose–response relationship such that greater DOE has been generally linked with greater risk to children's mental health (Kelley et al. 2010; Masten and Narayan 2012). Critically, however, although evidence from the COVID‐19 pandemic has revealed similar findings (i.e., the more the child was exposed, the more they exhibited mental health symptoms) (e.g., Shorer and Leibovich 2020), research suggests that the dose–response relationship may significantly vary as a function of environmental influences. Specifically, as detailed in the sections below, concurrent caregiving experienced by the child in real time (i.e., during the pandemic) was found to exert both moderating (e.g., Cohodes et al. 2021) and mediating effects (e.g., Shorer and Leibovich 2020; Spinelli et al. 2020). In the present report, we sought to shed further light on such moderating and mediating effects of the immediate caregiving environment. Moreover, the current study adds to previous studies by its examination of whether, beyond the effects of concurrent caregiving, *previous* caregiving, experienced by the child as early as infancy, several years *prior* to the stressor onset, may also have exerted moderating effects in the face of the future large‐scale stressor. Such findings would be in line with previous research revealing that *early* caregiving factors experienced in infancy foster resilience and lay the foundations for long‐lasting positive outcomes for children (Calkins and Hill 2007; Morales and Fox 2019; Morris et al. 2017). Although a few studies have demonstrated longitudinal links between caregiving experienced prior to the onset of a large‐scale stressor and children's future emotional responses (Achterberg et al. 2021; Costa et al. 2009; Kessel et al. 2019), no study has directly examined whether infant caregiving moderates a child's future vulnerability to DOE when faced with a large‐scale stressor.

Taken together, in the present study we examined both moderating and mediating effects of the concurrent caregiving environment, as well as moderating effects of sensitive caregiving provided in early infancy, several years prior to stressor onset. To the best of our knowledge, the present study is the first to simultaneously assess both concurrent “real‐time” caregiving effects along with moderating longitudinal caregiving effects. In doing so, we shed light on the complex interplay and unique contribution of each effect in shaping child outcomes.

## 1 Concurrent Influences of the Caregiving Environment in the Context of Large‐Scale Stressors

When considering the variation in young children's emotional responses to the pandemic, it is important to keep in mind that caregiving factors may exert significant effects in *real time*, when the child is enduring the stressful event. Studies have revealed that for very young children, adaptive responses to large‐scale stressors significantly associate with environmental factors, such as caregiver's mental health and level of distress (Chemtob et al. 2010) as well as caregiver's behavior (Comer et al. 2008; Scheeringa et al. 2004; Williamson et al. 2017). As detailed below, previous literature points to both moderating and mediating effects of the immediate caregiving environment.

### 1.1 Evidence Suggesting Mediating Effects of Real‐Time Caregiving

Large‐scale stressors afflict not only young children but their caregivers as well, often compromising caregivers' ability to provide the positive environment necessary for optimal child adjustment. Evidence from the COVID‐19 pandemic has demonstrated clinical levels of parental distress arising from the demands of child‐rearing in pandemic‐related chaotic and irregular circumstances (for systematic review and meta‐analysis, see Racine et al. 2022), especially at the peak of stay‐at‐home mandates (i.e., lockdown; Adams et al. 2021). Heightened parental distress may negatively impact parenting behaviors. Indeed, during lockdowns parents reported engaging in poorer parenting behaviors and attitudes, such as potential child abuse, harsh parenting (Brown et al. 2020a; Chung et al. 2020), lower levels of closeness to their children, and greater parent–child conflict (Chung et al. 2020; Russell et al. 2020).

Critically, research on previous large‐scale stressors and disasters has revealed that the quality of caregiving, assessed concurrently, may play a central role in helping children adapt to the stressful experience (for reviews, see Cobham et al. 2016; Masten and Narayan 2012). Research has shown that the increased levels of parental distress and the subsequent decline in parenting quality due to pandemic‐related stressors, in turn, were associated with children's mental health (for reviews and meta‐analysis, see Chung et al. 2024; Stracke et al. 2023).

Although the mediating role of caregiving has rarely been directly examined, several findings appear to suggest a potential mechanistic mediating pathway of risk going from DOE, through caregiver distress and behavior, to child symptoms. Such studies have explored the direct associations between caregivers' mental health, level of distress, and behavior, and children's stress responses during the ongoing experience of the stressful event, or immediately thereafter (for review, see Cobham et al. 2016). For example, after Hurricane Katrina, caregivers' self‐reported distress was associated with levels of post‐traumatic symptoms among their 7–10 year‐old children (Gil‐Rivas et al. 2010). Similarly, after 9/11 maternal depression and PTSD were found to be significantly associated with increased behavioral problems, emotional reactivity, and aggressive behavior in preschool children, as reported by both mothers and teachers (Chemtob et al. 2010). After the 2004 tsunami, parents' self‐reported better functioning (e.g., decreased sick leave) was associated with higher levels of child adjustment in children ages 6–17 years (Hafstad et al. 2010). Similarly, better parent–child relationship quality was associated with decreased PTSD and depression in adolescents (ages 12–19 years) after tsunami exposure (Wickrama and Kaspar 2007). Relatedly, children (ages 7–13 years) discussing terrorism‐related news with their mothers coped better when their mothers were trained in how to calmly discuss the events, compared to a group where “discussion as usual” took place (Comer et al. 2008). Finally, Turkish earthquake‐exposed children (ages 7–14 years) reported higher post‐traumatic stress in relation to parents' reported irritability and detachment symptoms (Kiliç et al. 2003). In sum, parents' own levels of stress and behavior practices, when enduring a large‐scale stressor, appear to have meaningful links with children's mental health symptoms.

In the context of the COVID‐19 pandemic, a systematic review and a meta‐analysis (Stracke et al. 2023) reported direct associations between parental mental health symptoms and child mental health outcomes during the pandemic, with the largest effects found for parenting stress. The review further emphasized that dysfunctional parent–child interactions appeared to be a crucial mechanism for the transmission of mental health symptoms. Finally, one study directly examined the mediating role of concurrent parental distress in underlying indirect links between exposure to pandemic‐related stressors and child outcomes. Results revealed that high parental stress significantly mediated detrimental impacts of the pandemic‐related stressors on young children's mental health (Spinelli et al. 2020). Similarly, Shorer and Leibovich (2020) revealed that parental emotion regulation mediated the relationship between child's exposure to pandemic‐related stressors and a child's stress reactions.

### 1.2 Evidence Suggesting Moderating Effects of Real‐Time Caregiving

The protective moderating role of concurrent caregiving in shaping a child's stress responses has been supported by studies on child stress regulation, showing that the presence, responsiveness, and support of a caregiver can attenuate the associations between the impact of a stressful situation and a child's stress reactivity, in both naturalistic and laboratory settings (Albers et al. 2008; Brown et al. 2020b; Cobham et al. 2016; Feldman et al. 2010; Roberts et al. 2015; Valiente et al. 2004; Wolchik et al. 2000). In the context of large‐scale stressors, parents' emotional state and behavior also appear to exert significant moderating effects. For example, parental overprotectiveness was shown to amplify the positive association between exposure to a major flood in Poland and adolescents' (mean age 16) PTSD symptoms, whereas low parental overprotectiveness buffered this link (Bokszczanin 2008). Similarly, in the context of the COVID‐19 pandemic, higher parenting stress and anxiety amplified the association between pandemic‐related stressors (e.g., unemployment, financial insecurity) and children's internalizing and externalizing symptoms (Cohodes et al. 2021). Although most studies found an amplification effect in the link between exposure and child's emotional symptoms, one study found an inverse moderation pattern in the aftermath of Hurricane Katrina. Specifically, Spell et al. (2008) examined children aged 8–16 and found that the extent of hurricane exposure positively predicted children's internalizing symptoms when mothers reported lower levels of distress, but not when they reported higher levels of distress. In interpreting these unexpected findings, the authors suggested that mothers' psychological distress was positively linked with children's symptoms, irrespective of their level of hurricane exposure. In contrast, healthy maternal adjustment (characterized by lower levels of maternal distress) may have served as a protective factor, particularly for children with low disaster exposure. This interpretation is consistent with general child development research, which consistently shows that maternal mental health is a strong predictor of child mental health (e.g., Chen et al. 2020), regardless of exposure to other adversities. Although the results of Spell et al. (2008) suggest that healthy maternal adjustment may act as a protective factor for children with lower disaster exposure but not necessarily for those with higher exposure, the majority of studies have found that high maternal distress amplifies the impact of high exposure, leaving children more vulnerable (Bokszczanin 2008; Cohodes et al. 2021; Dubow et al. 2012; Dyb et al. 2011). Inconsistent findings may be due to factors such as differences in exposure levels, the nature of the stressor, child's age, or methodological approaches, which can influence how maternal distress moderates child outcomes.

## 2 Early Influences of the Caregiving Environment Provided Prior to Stressor Onset

Abundant literature has demonstrated the importance of the early caregiving environment in shaping children's long‐lasting vulnerability versus resilience. The “stress sensitization model” posits that early stressful caregiving experiences are longitudinally linked with children's future vulnerability to later stressful events (McLaughlin et al. 2015; Wade et al. 2019, 2020). Likewise, optimal early caregiving experiences have been found to associate with the development of children's emotional regulatory skills (Calkins and Hill 2007; Morales and Fox 2019; Morris et al. 2017), which support adaptive responses to everyday stressors (e.g., Flouri et al. 2014; Troy and Mauss 2011). Developmental neuroscience has established infancy as a particularly sensitive time window during which caregiver‐infant interactions shape the development of the infant's brain (Nelson et al. 2014) as well as neural structures supportive of optimal stress regulation (Hane and Fox 2006; Schore 2001). Attachment theory and research (e.g., Bowlby 1969; Smyke et al. 2010), as well as current neuroscience perspectives on attachment (Schore and Schore 2008), mark the first year of life as the unique time window during which infant attachment styles and associated long‐lasting regulatory patterns are crystalized.

A prominent caregiving factor found to play a key role in supporting the development of secure attachment (for review, see Belsky and Fearon 2016) and associated stress responses (e.g., Abraham et al. 2021; Hane and Fox 2006) is “maternal sensitivity.” Maternal sensitivity refers to the caregiver's ability to notice the child's signals and respond to them promptly and appropriately (Ainsworth et al. 1974), providing appropriate vocal, physical, and affective stimulation. Maternal sensitivity is commonly coded from observations of dyadic interactions between mother and child, indexed by maternal responsiveness and acknowledgment of child cues, supportive presence, and warm appropriate range of affect (for systematic review, see Mesman and Emmen 2013).

A rich body of findings has demonstrated that variations in maternal sensitive behavior in the context of daily activities are linked with individual differences in neural and physiological markers of children's stress regulation (Asok et al. 2013; Garnett et al. 2020; Gunnar 2006; Hane and Fox 2006). Based on these findings, theory posits that the caregiver's sensitive responding to the infant's needs serves as an external regulator (Crockenberg and Leerkes 2004), which eventually associates with the child's emerging capacity to self‐regulate (Bernier et al. 2010). It is not surprising that the attachment system is often thought of as a regulatory system (Schore and Schore 2008). The infant's early experience within the attachment relationship is thought to shape their developing regulatory capacities. As such, one would predict that the experience of early sensitive caregiving would foster future resilience and thereby attenuate the child's vulnerability when exposed to a large‐scale stressor.

A few studies have examined the links between children's early caregiving, experienced prior to the onset of a large‐scale stressor, and children's future emotional responses to large‐scale stressors (Achterberg et al. 2021; Costa et al. 2009; Kessel et al. 2019). For example, a study that assessed youth (ages 6–17) pre‐and post‐Hurricane Katrina found that lower attachment‐related parenting magnified the stability of adolescents' anxiety from before Hurricane Katrina to after it (Costa et al. 2009). In the context of the COVID‐19 pandemic, Achterberg et al. (2021) found that parental over‐reactivity reported by parents prior to the pandemic (i.e., parental irritability, anger, frustration, and tendencies toward harsh parenting) was associated with increased stress responses to COVID‐19 lockdowns among children ages 10–13 years. Surprisingly, no study has directly examined whether sensitive infant caregiving moderates children's future vulnerability in the face of large‐scale stressors (i.e., whether it moderates the links between DOE and child outcomes).

## 3 Current Study

In the current study, we aimed to assess both concurrent and early influences of the caregiving environment to examine the unique contribution of each influence to children's internalizing and externalizing symptom responses to the large‐scale stressor of the COVID‐19 pandemic. Importantly, whereas previous research relied solely on parent‐reported measures to assess caregiver influences during the pandemic, we examined both self‐reported measures of maternal stress as well as behavioral observations of maternal behavior videorecorded remotely during structured mother–child interactions. Specifically, we assessed the mediating and moderating effects of concurrent maternal stress and behavior during a nationwide lockdown, and we assessed the moderating effects of early caregiving behavior observed during infancy. We report on a sample of preschoolers assessed during a pandemic‐related nationwide lockdown in Israel, whom we had previously assessed during infancy at the age of 4 months, several years prior to the pandemic outbreak.

With regard to the potential mediating effects of concurrent caregiving, the mediating influences of concurrent caregiver stress and behavior might be particularly amplified during a lockdown, when additional adults are less accessible to the child. Lockdowns themselves may amplify variability in caregivers' stress and behavior (Adams et al. 2021), thus making lockdowns a particularly ideal time window for assessing the role of real‐time caregiving in the underlying links between pandemic‐related stressors and children's stress responses. We assessed the indirect link between child's DOE and child's internalizing and externalizing symptoms through maternal stress and behavior. Specifically, we expected that child's DOE would be positively linked to maternal stress, which in turn would be positively linked to child's internalizing and externalizing symptoms. We also expected that child's DOE would be negatively linked to maternal sensitive behavior during the lockdown, which in turn would be negatively linked to child's internalizing and externalizing symptoms. For both pathways, we expected to find significant indirect effects between child's DOE and child's internalizing/externalizing symptoms.

With regard to moderating effects, we examined whether a child's vulnerability to the detrimental effect of DOE may have varied as a function of maternal‐reported stress and/or level of maternal sensitive behavior assessed during the lockdown. In other words, we explored whether maternal stress and behavior assessed during the lockdown moderated associations between child's DOE and a child's internalizing and externalizing symptoms. At high levels of maternal‐reported stress during the lockdown, we expected to find significant links between child's DOE and a child's internalizing and externalizing symptoms, whereas at low levels of maternal stress, we expected this DOE‐related risk to be attenuated. Similarly, at low levels of maternal sensitivity observed during the lockdown, we expected to find significant links between child's DOE and a child's internalizing and externalizing symptoms, whereas at high levels of maternal sensitive behavior, we expected this DOE‐related risk to be attenuated.

We further propose that the pandemic offers an ideal study context for the potential identification of early preventive caregiving influences. We posit that in addition to concurrent caregiver effects, caregiving experienced *prior* to stressor onset, as early as infancy, might be particularly key in moderating future child outcomes. Due to methodological challenges, studies on child resilience to large‐scale stressors rarely have access to pre‐stressor data, let alone early caregiving experienced during the formative time window of infancy. We propose that early maternal sensitive care, provided during the important time window of infancy, several years prior to pandemic onset, may have foundationally attenuated vulnerability to mental health symptoms and fostered young children's future resilience when forced to endure the large‐scale stressor of the COVID‐19 pandemic.

We specifically assessed the extent to which normative variations in sensitive caregiving experienced in infancy, several years prior to the pandemic, moderated the child's future vulnerability to DOE. Critically, when examining potential moderating effects of infant caregiving, we controlled for the effects of concurrent maternal stress and behavior assessed during the lockdown. At low levels of maternal sensitivity observed in infancy, we expected to find significant links between child's DOE and the child's internalizing and externalizing symptoms, whereas at high levels of infant maternal sensitive care, we expected this DOE‐related risk to be attenuated.

## 4 Methods

### 4.1 Participants

Two‐hundred‐and‐fifty‐eight participants were recruited toward the end of their pregnancies from a major hospital in Israel. Participants were recruited as part of a larger study on the childbirth experience. Exclusion criteria at the time of recruitment were multiple pregnancy or serious medical issues. When infants reached the age of 4 months, mothers were invited to participate in an assessment conducted at their home (*N*_time point 1_ = 147; *M*_age_ = 4.33 months, SD = 0.60). Data collection took place between January 2016 and December 2017. A few years later, following the COVID‐19 pandemic outbreak, during a nationwide lockdown, participants were invited to participate in a follow‐up assessment which was conducted remotely. Of the initially recruited 258 participants, 186 consented to participate in the follow‐up assessment. Data collection took place between October and December 2020, and at this time children's ages ranged between 36 and 60 months (*N*_time point 2_ = 186; *M*_age_ = 49.26 months; SD = 5.97). The present study includes participants who participated at both time points (*N* = 133), only at the first time point (*N* = 14), or only at the second time point (*N* = 53). Data analyses in the present study were conducted on participants who had available data for at least one of the two study time points (*N* = 200). At the time of recruitment, mothers ranged in age from 20 to 44 years (*M* = 31.83, SD = 4.01) and had between 10 and 22 years of education (*M* = 15.27, SD = 2.39). The majority of mothers (51.8%) reported a monthly household income of 4000–7500 US dollars; 63.5% were secular Jews; 53.5% were primipara; and 50% of the expected births were female. Handling of missing data is described in the data analytic section below.

### 4.2 Measures

#### 4.2.1 Sociodemographic Covariates

At the time of recruitment, mothers were asked to report demographic characteristics including maternal education, maternal and child age, family income, number of children, sex of the expected infant, and religion. At T2, during the pandemic, mothers were asked to update the information on their age, their child's age, and the number of children in the family.

#### 4.2.2 Early Maternal Sensitive Caregiving During Infancy (T1)

Characteristics of maternal sensitive caregiving behavior within observed mother‐infant dyadic interactions were assessed in the participants' homes at 4‐month postpartum. A five‐minute mother‐infant free‐play interaction was videorecorded and coded offline by two experienced coders using the Coding Interactive Behavior (CIB) coding scheme (Feldman 1998), on a 5‐point Likert scale. In line with Feldman et al. (2009), maternal sensitive caregiving scores comprised eight CIB subscales: maternal acknowledgement of and responsiveness to child's communication; warm vocal quality; continuous gaze; appropriate range of affect; resourcefulness; consistency of style; supportive presence; and adaptation to infant signals. Double coding of 23% of the sample yielded moderate inter‐rater reliability; two‐way random, absolute agreement ICC = 0.73.

#### 4.2.3 Dose of Exposure (DOE) to Pandemic‐Related Psychosocial Stressors (T2)

This measure (child's DOE) was used to assess individual differences in the extent to which the children were exposed to (i.e., experienced) various objective psychosocial stressors reported to be prevalent during the time of the pandemic. In other words, it served to measure the number and severity of pandemic‐related hardships accumulated for each child, potentially impacting the child's emotional state. Child's DOE was assessed using a slightly adapted version of the 6‐item exposure scale employed by Shorer and Leibovich (2020), indexing exposure to pandemic‐related psychosocial stressors. Mothers indicated the extent to which their child was exposed to seven specific pandemic‐related stressors (i.e., having a close acquaintance who was sick with COVID‐19; being closely acquainted with a person in quarantine; being closely acquainted with a person who permanently lost their job; being closely acquainted with a person who temporarily lost their job; receiving disturbing information on COVID‐19 via the media; being confined to home; obtaining explanations regarding COVID‐19) on a 3‐point scale (1 = no exposure, 2 = some exposure, and 3 = extensive exposure). Items were averaged and scored to create a total pandemic DOE score. The overall internal consistency was α = 0.66, which reflects low to moderate reliability. Notably, low to moderate internal consistency is common for indices of experienced life events, as these measures often reflect high variability due to the diverse and individualized nature of life events, which can differ significantly across individuals and contexts.

#### 4.2.4 Maternal Stress During Lockdown (T2)

The *Parenting Stress Index‐Short Form* (Abidin 2012) is a 36‐item questionnaire that evaluates the magnitude of stress in the parent–child system. Using a 5‐point Likert scale from 1 (strongly agree) to 5 (strongly disagree), mothers indicate the extent to which they agree or disagree with statements reflecting parent‐related stress (e.g., “I feel trapped by my responsibilities as a parent”). Items are reversed so that higher scores indicate higher parenting stress. A total stress score was calculated in line with standard scoring (Abidin 2012). Raw scores of 86–90 are considered “high stress,” and raw scores above 91 are considered “clinical levels” of stress. The overall internal reliability was *α* = 0.92.

#### 4.2.5 Maternal Sensitive Caregiving During Lockdown (T2)

Maternal behavior was videorecorded during the mother's structured play with her child in the dyadic *Etch‐A‐Sketch Online* (ESO) task, an internet‐enabled “onscreen” Etch‐a‐Sketch controlled via keyboard. The structured play sessions were coded using the Parent–Child Interaction System (PARCHISY), commonly employed for coding the ESO task (Deater‐Deckard et al. 1997). Each scale was rated using a 7‐point scale ranging from 1 (no instances) to 7 (constant evidence) of the relevant behaviors for each scale. Maternal sensitive caregiving scores comprised two scales: maternal positive affect and maternal responsiveness. The two behaviors were correlated (*r =* 0.54, *p* ≤ 0.001) and thus standardized and averaged into a single composite. Double coding of 17% of the sample yielded excellent inter‐rater reliability; two‐way random, absolute agreement ICC = 0.94.

#### 4.2.6 Child's Internalizing and Externalizing Symptoms During Lockdown (T2)

The *Child Behavior Checklist*/*1.5–5* (Achenbach and Leslie 2000) is a standard measure of child behavioral and emotional problems across multiple dimensions (e.g., attention problems, aggressive behavior, depression) suitable for preschool children. Mothers were asked to rate the frequency of the manifestations of their child's behavior during lockdown with regard to 100 items, using a 3‐point scale (0 = not true; 1 = somewhat true or sometimes true; 2 = very true or often true). Two symptom scales—internalizing (anxiety/depression, social withdrawal, emotional reactivity, somatic complaints) and externalizing (aggressive behavior, hyperactivity, and inattention)—were computed based on the ratings. In line with standard recommendations (Achenbach 1991), raw scores were employed for the purpose of assessing individual differences of children's symptoms. Raw scores capture subtle variations in behavior that might be lost when converting them to t‐scores, allowing for more variability which can help detect effects that might be obscured by the compression of scores into standardized units. Raw scores of 18 and 14 on the internalizing scale are the respective cut‐offs for clinical and subclinical levels of internalizing symptoms (equivalent to internalizing *t*‐scores of 64 and 60). Raw scores of 25 and 21 on the externalizing scale are the respective cut‐offs for clinical and subclinical levels of externalizing symptoms (equivalent to externalizing *t*‐scores of 64 and 60). Internal reliability was satisfactory for both scales (internalizing behavior: *α* = 0.89; externalizing behavior: *α* = 0.92).

### 4.3 Data Analytic Approach

Initial models were created based on theory, and covariates were included on the basis of significant correlations of demographic variables with main study variables. Given the theoretical basis and the a priori hypotheses, the analyses presented are relatively confirmatory. Preliminary analyses assessed descriptive information, correlations between all study variables, and correlations between study variables and potential demographic covariates (see Table 2). Demographic variables that significantly correlated with study variables were included as covariates in subsequent analyses and were then removed if they did not significantly contribute to the model. Covariates were added to the model by regressing them on the relevant study variable with which they were found to correlate. Main analyses were performed using R package *lavaan* (Rosseel 2012) to estimate the model parameters and to compute model fit. All continuous variables were standardized.

Separate models were examined for each of the outcome variables: internalizing and externalizing symptoms (Models 1 and 2, respectively). For each model, analyses were conducted in two steps resulting in Models 1a and 1b predicting internalizing symptoms and 2a and 2b predicting externalizing symptoms. Specifically, the first step examined the effects of concurrent caregiving, simultaneously assessing both moderating and indirect effects of maternal stress and maternal sensitive behavior assessed in real time during the lockdown (Models 1a and 2a). The second step (Models 1b and 2b) examined the moderating role of pre‐pandemic caregiving (i.e., the moderating role of early maternal sensitive caregiving observed during infancy). Importantly, given the fact that pre‐pandemic caregiving was significantly longitudinally associated with concurrent caregiving, the moderating role of pre‐pandemic caregiving was examined while controlling for concurrent caregiving. Given the limitation in statistical power, a stepped model trimming approach was employed such that insignificant moderating and indirect effects of concurrent caregiving were trimmed in the second step based on *p*‐values as well as a comparison of model fit. Finally, all significant moderating effects were further investigated via simple slopes analysis conducted at 1 SD above and below the mean.

Model fit was assessed by means of the likelihood‐ratio test (*χ* ^2^), Root Mean Square Estimation of Association (RMSEA), the Bentler Comparative Fit Index (CFI), and standardized root mean square residual (SRMR) (Barrett 2007). Good model fit was indicated by a non‐significant *χ* ^2^, an RMSEA lower than 0.06, a CFI larger than 0.95, and SRMR values less than 0.08 (Hu and Bentler 2009).

Missing data for DOE (9%), maternal stress (7.5%), maternal observed sensitive behavior during lockdown (27%), child's internalizing and externalizing symptoms (10%), and maternal observed sensitive caregiving in infancy (26.5%) were handled by model estimation through full information maximum likelihood (FIML) estimation. Full information maximum likelihood estimation can be used under the assumption of “missing data completely at random,” which was tested by means of Little's MCAR test, and for which *p* > 0.05 indicates “missing data completely at random” (Little 1988). Analyses of the current data confirmed that missing data were missing completely at random (Little's MCAR tests: *χ* ^2^ = 25.53, *p* = 0.08).

Power analyses indicated that given a small to medium effect size (f2 = 0.10) and an error probability of 0.05, a sample of *n* = 184 was necessary to achieve 0.85 power for Models 1a and 2a and a sample of *n* = 168 for Models 1b and 2b.

### 4.4 Procedure

The study was approved by the IRB and mothers provided written informed consent. At 4‐months postpartum (T1), a researcher visited the participants in their homes. In line with standard protocol (Feldman 1998), after explaining the study procedures, the researcher asked the mother to play with the infant for five minutes “as she usually does.” At 36–60 months (T2), data were remotely collected during a nationwide lockdown between October and December 2020. It should be noted that during this lockdown all childcare centers were closed (i.e., all participants lacked access to external childcare), ensuring a similar context of parental burden in this aspect across the study sample. T2 assessments comprised online maternal‐report questionnaires and observed maternal sensitive caregiving during the lockdown. Sensitive caregiving behavior was coded offline from a mother–child structured‐play interaction which was videorecorded remotely during videoconferencing.

## 5 Results

### 5.1 Preliminary Analysis

Descriptive data revealed relatively high levels of children's internalizing and externalizing symptoms as well as maternal stress during the COVID‐19 pandemic. More specifically, mothers' reports regarding their children revealed clinical and subclinical levels of both internalizing symptoms (anxiety, depression, social withdrawal) and externalizing symptoms (aggressive behavior, hyperactivity, and inattention) presented by 15% and 13% of children, respectively. A Pearson chi‐square goodness‐of‐fit test showed that this prevalence was significantly higher than the expected norm prevalence of 7% (Achenbach and Leslie 2000), both for internalizing symptoms [*χ* ^*2*^ (1, 180) = 17.7, *p* < 0.001] and for externalizing symptoms [*χ* ^*2*^ (1, 180) = 7.54, *p* < 0.01] Maternal self‐reports regarding maternal stress revealed that 29.7% of mothers reported clinical levels of stress. A Pearson chi‐square goodness‐of‐fit test showed that this prevalence was significantly higher than the expected norm prevalence of 10% (Abidin 2012) [*χ* ^*2*^ (1, 185) = 80.02, *p* < 0.001].

Table 1 presents correlations between the study variables. The data reveal significant positive associations between preschooler's DOE and concurrent internalizing and externalizing symptoms. Moreover, higher levels of maternal stress were associated with child's greater number of internalizing and externalizing symptoms. Child's age was significantly negatively associated with child's externalizing symptoms and was thus included as a covariate in all subsequent analyses assessing externalizing symptoms.

**TABLE 1:** Descriptive statistics and correlations among study variables.

		M (SD)	*N*	1	2	3	4	5
1.	Child DOE (T2)	0.65 (0.40)	182	—				
2.	Child internalization (T2)	7.62 (7.55)	180	0.31 (180)***	—			
3.	Child externalization (T2)	10.64 (8.20)	180	0.24 (180)**	0.77 (180)***	—		
4.	Maternal stress (T2)	80.70 (18.41)	185	0.22 (182)**	0.60 (180)***	0.67 (180)***	—	
5.	Maternal sensitive caregiving during lockdown (T2)	5.02 (1.19)	146	−0.02 (145)	−0.05 (145)	−0.07 (145)	−0.04 (145)	—
6.	Maternal sensitive caregiving during infancy (T1)	33.90 (6.00)	147	−0.05 (132)	−0.12 (132)	−0.11 (132)	0.00 (132)	0.21 (115)*

*Note:* DOE Dose of exposure to COVID‐related psycho‐social stressors. *T1* data collected during infancy (4‐months old) *T2* data collected during nationwide lockdown imposed by COVID‐19 (36–60 months old).

* *p* < 0.05.

** *p* < 0.01.

*** *p* < 0.001.

As a next step, we assessed potential control variables from T1 (infant's age, maternal age, number of children in the family, infant's sex, maternal education, household income, and religiousness) as well as from T2 (child's age, maternal age, and number of children in the family). As presented in Table 2, significant correlations were found between child's DOE and maternal education, household income, as well as maternal age during the pandemic (T2). Additionally, maternal sensitive behavior during the pandemic (T2) was correlated with maternal education, household income, and religiousness. Finally, significant correlations emerged between maternal sensitive behavior during infancy (T1) and maternal age (T1) and number of children (T1). As such, these variables were considered to be covariates in subsequent analyses (by regressing it on relevant variables).

**TABLE 2:** Descriptive statistics and correlations between study variables and demographic variables.

	*N*	M (SD)	SD	Maternal sensitive behavior (T1)	Child DOE (T2)	Maternal stress (T2)	Maternal sensitive behavior (T2)	Child internalizing symptoms (T2)	Child externalizing symptoms (T2)
Infant age (Month; T1)	147	4.35	0.55	−0.11	—	—	—	—	—
Maternal age (T1)	147	32.48	3.95	0.22**	—	—	—	—	—
Number of children (T1)	200	1.67	0.92	0.19*	—	—	—	—	—
Maternal education (T1)	197	15.26	2.39	0.22**	−0.15*	0.01	0.25**	−0.08	−0.08
Child age (T2)	183	4.10	0.50	—	0.03	−0.08	−0.06	−0.07	−0.15*
Maternal age (T2)	183	35.98	4.11	—	−0.18*	−0.03	0.01	−0.14	−0.08
Number of children (T2)	186	2.38	0.85	—	−0.12	−0.10	−0.08	−0.15	−0.12

	*N*	%	*t*/*F*	df	*t*/*F*	df	*t*/*F*	df	*t*/*F*	df	*t*/*F*	df	*t*/*F*	df
**Child sex (T1)**	200		1.18	144	−0.53	180	−1.40	183	0.37	144	−0.72	178	−0.97	178
Male		50.0%												
**Household income (T1)**	189		2.05	4	3.30*	**4**	0.76	4	2.51*	**4**	2.23	4	1.34	4
0–6000 NIS		10.0%												
6000–12,000		20.0%												
12,000–18,000		24.5%												
18,000–24,000		24.5%												
Above 24,000		15.5%												
**Religiousness**	197		1.72	3	0.75	3	0.89	3	4.09**	**3**	1.97	3	1.56	3
Secular		63.5%												
Traditional		20.0%												
Religious		12.5%												
Very religious		2.5%												

*Note:* *p < 0.05, **p < 0.01.

### 5.2 Step 1—Moderating Effects of Maternal Variables Assessed During the Lockdown

**FIGURE 1:**
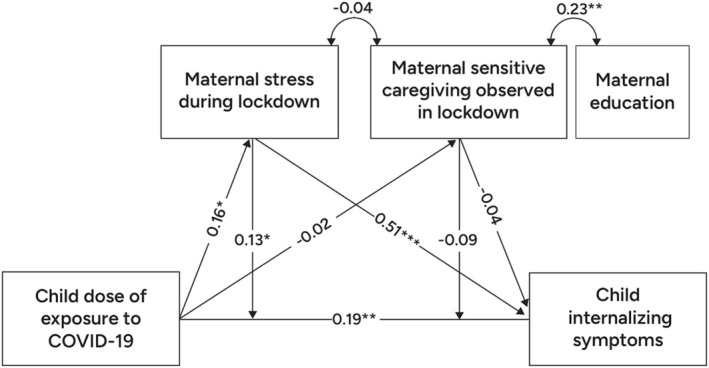
Path Analysis Model of associations between child exposure to Covid‐related psycho‐social stressors, child internalizing symptoms, and parenting during lockdown. Coefficients presented are standardized parameter estimates. Model fit: *χ* ^2^(9) = 8.21, *p* = 0.51; RMSEA = 0.00, CFI = 1.00; SRMR = 0.04. *p < 0.05, **p < 0.01, ***p < 0.001.

Path analysis tested two proposed models. The first model (Model 1a), predicting internalizing symptoms, is depicted in Figure 1. Analyses revealed that preschoolers' increased display of internalizing symptoms during the lockdown was significantly predicted by both child's elevated exposure to pandemic‐related psychosocial stressors (DOE) (*β* = 0.19, *p* < 0.01, 95% CI 0.071; 0.327) and elevated maternal stress during the lockdown (*β* = 0.51, *p* < 0.001, 95% CI 0.414; 0.638). Furthermore, a significant indirect path emerged from the child's DOE to child's internalizing symptoms, through concurrent maternal‐reported stress (*β* = 0.08, *z* = 1.98, *p* < 0.05, 95% CI 0.004; 0.172). Finally, maternal stress during the lockdown significantly moderated the links between the child's DOE and concurrent internalizing symptoms (*β* = 0.13, *p* < 0.05, 95% CI 0.006; 0.258). No such indirect paths or moderating effects were found for maternal sensitive caregiving behavior observed during the lockdown.

**FIGURE 2:**
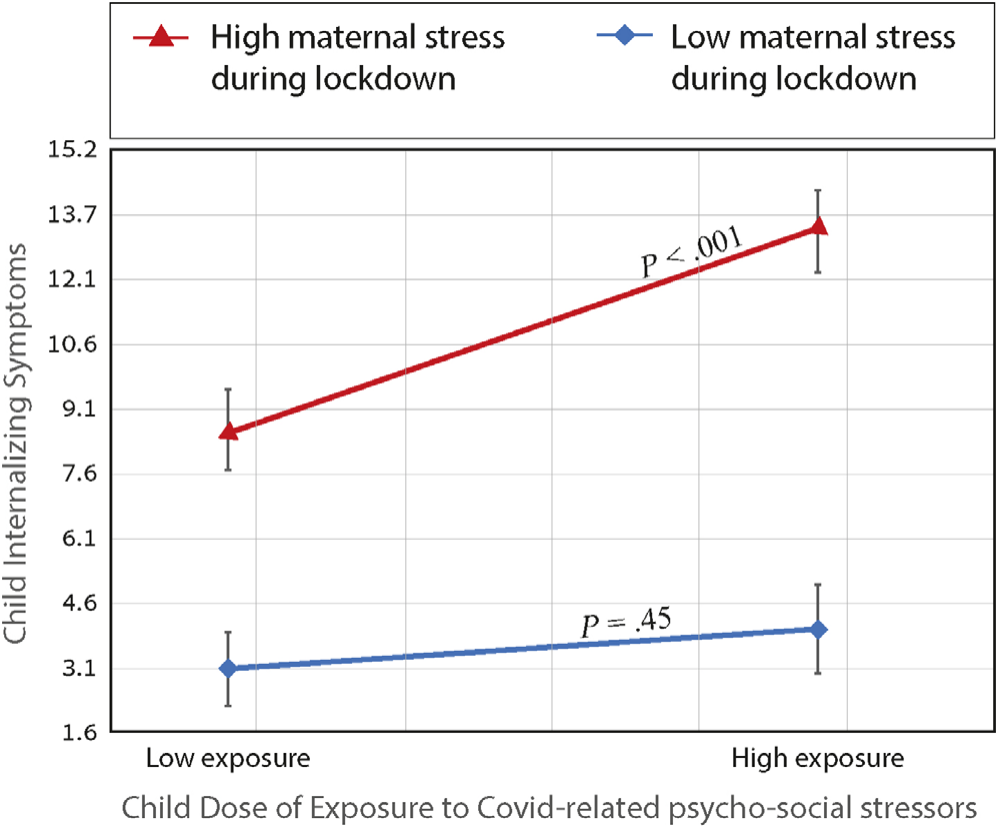
Simple Slopes of Maternal Stress during Lockdown Moderation Effect. The conditional effect of exposure to Covid‐related psycho‐social stressors was significant only when maternal stress during lockdown was high.

Follow‐up analyses for the moderating effect of maternal‐reported stress (simple slopes 1 SD above and below the mean) revealed that at high levels of maternal stress, preschoolers displayed increased vulnerability to pandemic‐related stressors—namely, the child's higher DOE was associated with child's elevated internalizing symptoms (*β* = 0.32, *p* < 0.001, 95% CI 0.154; 0.510). However, at low levels of maternal stress, there was no significant link between the child's DOE and child's internalizing symptomology (*β* = 0.07, *p* = 0.45, 95% CI −0.110; 0.254). See Figure 2 for a depiction of the differential effects of the child's DOE on child's internalizing symptoms as a function of high versus low levels of maternal stress during the lockdown.

The second model for predicting externalizing symptoms (Model 2a) revealed that the child's increased display of externalizing symptoms during the lockdown was significantly predicted by both child's elevated DOE (*β* = 0.12, *p* < 0.05, 95% CI 0.002; 0.237) and elevated maternal stress during the lockdown (*β* = 0.69, *p* < 0.001, 95% CI 0.585; 0.762). Furthermore, a significant indirect path emerged from the child's DOE to child's externalizing symptoms, through concurrent maternal‐reported stress (*β* = 0.11, *z* = 2.02, *p* < 0.05, 95% CI 0.007; 0.219). However, the link between the child's DOE and child's externalizing symptoms was not moderated by maternal‐reported stress during the lockdown (*β* = 0.01, *p* = 0.88, 95% CI −0.107; 0.124), nor by maternal sensitive caregiving observed during the lockdown (*β* = −0.01, *p* = 0.88, 95% CI −0.127; 0.109).

### 5.3 Step 2—Moderating Effects of Maternal Sensitive Caregiving Observed During Infancy

Path analysis tested two proposed models to examine the moderating role of maternal sensitive caregiving, observed during infancy, in addition to the moderating and indirect effects of maternal stress reported during the lockdown. In line with our stepped model trimming approach, moderating and indirect influences of maternal sensitive caregiving observed during the lockdown were not examined in the model, due to insignificant effects found in Step 1. Importantly, as indicated by preliminary analyses, maternal sensitive caregiving observed in infancy was significantly longitudinally associated with later maternal sensitive caregiving observed during the lockdown and was thus controlled for in all analyses.

**FIGURE 3:**
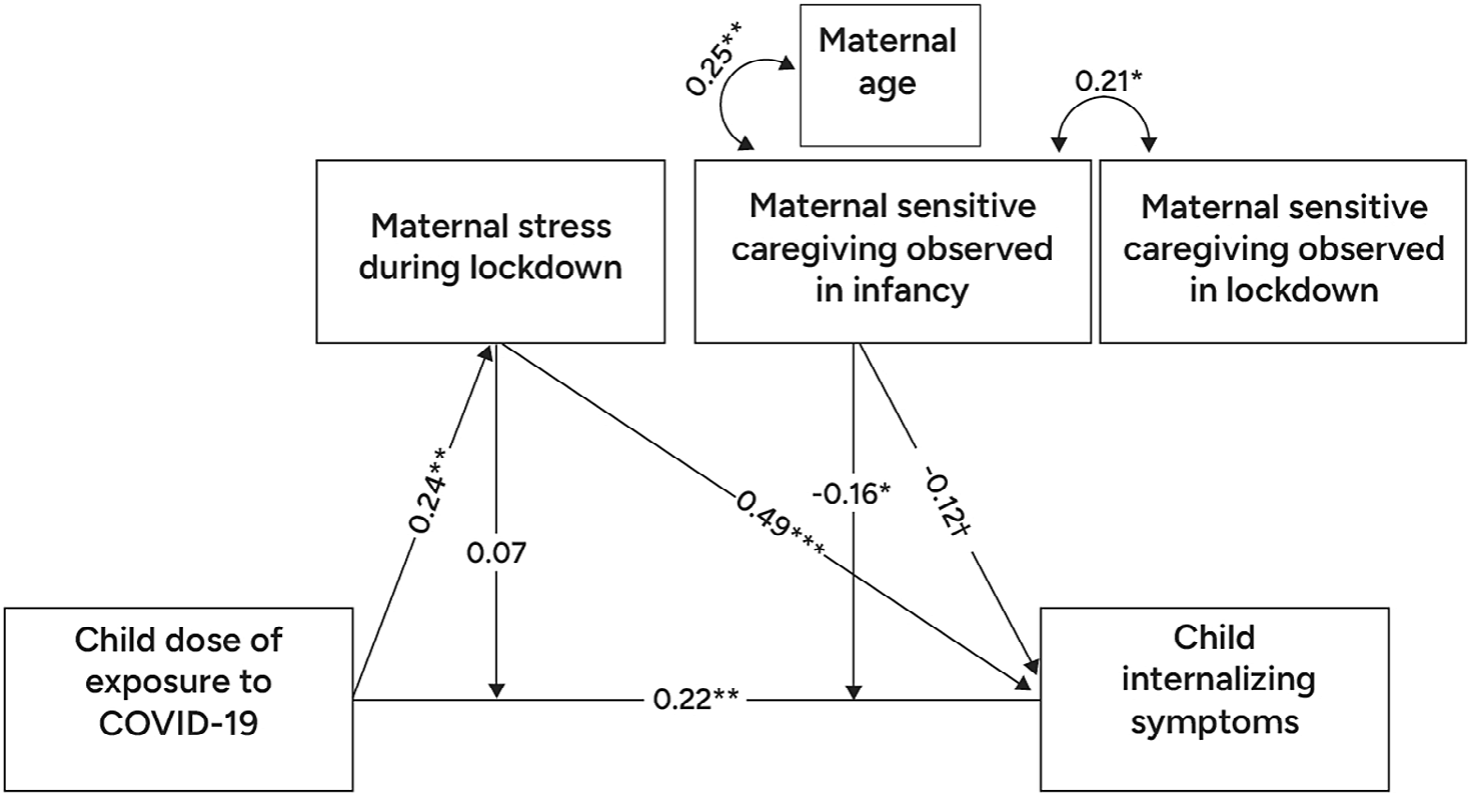
Path Analysis Model of associations between child exposure to Covid‐related psycho‐social stressors, child internalizing symptoms, and maternal sensitive caregiving during infancy. Coefficients presented are standardized parameter estimates. Model fit: *χ* ^2^(17) = 18.26, *p* = 0.37; RMSEA = 0.02, CFI = 0.98; SRMR = 0.06. ^†^ *p* < 0.10, *p < 0.05, **p < 0.01, ***p < 0.001.

For predicting internalizing symptoms (Model 1b), analyses revealed a significant moderating effect of sensitive maternal caregiving observed in infancy, several years prior to the pandemic onset (*β* = −0.16, *p* < 0.05; 95% CI −0.283; −0.011). Interestingly, once accounting for caregiving experienced during infancy, the moderating effects of maternal‐reported stress during lockdown were no longer significant. See Figure 3 for a depiction of the entire model.

**FIGURE 4:**
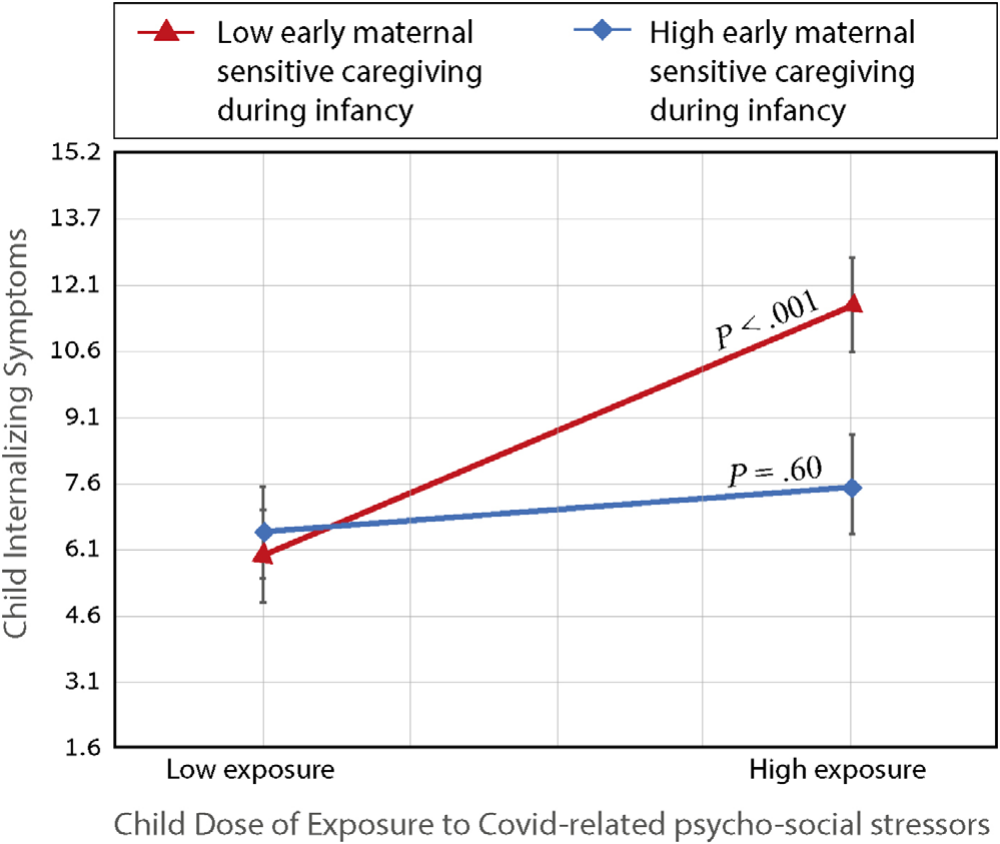
Simple Slopes of Maternal Sensitive caregiving during Infancy Moderation Effect. The conditional effect of exposure to Covid‐related psycho‐social stressors was significant only when levels of early maternal sensitive caregiving observed in infancy were low.

Follow‐up analyses of the moderation effect (simple slopes 1 SD above and below the mean) revealed that preschool children who experienced relatively lower levels of maternal sensitive caregiving during infancy were significantly vulnerable to pandemic‐related stressors several years later, displaying increased internalizing symptoms in response to high levels of exposure (*β* = 0.37, *p* < 0.001; 95% CI 0.178; 0.536). However, children who experienced high levels of maternal sensitive caregiving during infancy were not significantly vulnerable to pandemic‐related stressors several years later. At high levels of maternal sensitive caregiving experienced during infancy, pandemic‐related stressors exerted no significant impact on children's internalizing symptomology (*β* = 0.06, *p* = 0.60, 95% CI −0.147; 0.274). See Figure 4 for a depiction of the differential effects of child's DOE on child's internalizing symptoms as a function of high versus low levels of maternal sensitive caregiving observed in infancy.

In Model 2b, no such moderating effects of observed infant sensitive care emerged for the prediction of preschoolers' externalizing symptoms (*β* = −0.06, *p* = 0.43, 95% CI −0.183; 0.078).

## 6 Discussion

The present findings are in line with an increased number of studies evidencing the negative impact of pandemic‐related stressors on both children's mental health and their caregivers (Nearchou et al. 2020; Panchal et al. 2021; Racine et al. 2022). Taken together, the findings of the present study point to the significant roles of both the immediate caregiving environment and the early caregiving provided during infancy in shaping children's emotional vulnerability to large‐scale stressors.

In line with previous literature underscoring the important role of the immediate caregiving environment, the results of the current study demonstrate that children's mental health symptoms are susceptible to caregiver stress in real time. Specifically, after accounting for early maternal sensitivity provided during infancy, the direct effect of concurrent maternal stress on children's symptoms during the pandemic lockdown remained significant, as did the indirect associations between preschooler's DOE and preschooler's internalizing symptoms, significantly mediated by maternal‐reported stress during the lockdown (these significant effects can be seen in Figure 3). As such, maternal stress in real time seems to be an important mediating factor to target in clinical practice: the theoretical premise being that the presence and availability of a relatively non‐stressed caregiver may provide co‐regulation in the context of threat (Lionetti et al. 2022; Pat‐Horenczyk et al. 2015; Tang et al. 2021). These findings underscore the need to provide caregivers with the necessary support and means to reduce parental stress in the context of large‐scale stressors. The findings raise the possibility that a reduction of parental stress may be important not only for the parents' well‐being but also for supporting their capacity to provide the stress‐buffering necessary for the well‐being of their young children.

Moreover, the present study is the first to directly demonstrate that maternal sensitivity during infancy moderated the future pandemic‐related risk experienced several years later. These findings suggest that maternal sensitivity provided during the formative time window of infancy may lay the foundation for a preschooler's resilience in the face of future large‐scale stressors. Specifically, early exposure to sensitive maternal care during infancy significantly moderated the future associations between preschoolers' DOE and their concurrent internalizing symptoms reported during a pandemic lockdown (the significant moderation effect is depicted in Figure 4). Interestingly, when accounting for early maternal sensitivity, although the direct and indirect effect of concurrent stress remained significant, the *moderating* effect of concurrent maternal stress on the link between the child's DOE and child's outcome became nonsignificant. Moreover, protective moderating effects of early infant care remained significant after controlling for concurrent effects of real‐time caregiving behavior and stress assessed during the lockdown. *These findings point to the unique protective effect of early sensitive care, which is maintained even in the context of current heightened stress among caregivers*. Although previous studies established the long‐term stress sensitizing effects of early infant neglect, the current study is the first to directly demonstrate that normative variations in sensitive parenting may significantly attenuate a preschooler's stress vulnerability when faced with a future large‐scale stressor.

Taken together, the present results support the notion that comprehensive practical approaches should take into account both the potential buffering effect of early maternal sensitivity as well as the potential risk imposed by concurrent maternal stress during stressful times such as the COVID‐19 pandemic. As such, comprehensive approaches should target both early and concurrent caregiving.

### 6.1 Limitations and Future Directions

Although the present data revealed moderating effects of early infant care, it is important to emphasize that the current study design did not provide insights regarding the mechanisms which may have underlay these early moderating caregiving effects. Previous research suggests that optimal early caregiving experiences may foster resilience through children's development of emotional regulatory skills (Calkins and Hill 2007; Morales and Fox 2019; Morris et al. 2017), which support adaptive responses to everyday stressors (e.g., Flouri et al. 2014; Troy and Mauss 2011). Theory also suggests that sensitive early caregiving may instill a sense of security and trust (e.g., Bowlby 2008), which may be critical when children experience uncertainty and risk. The findings of the present study appear to be conceptually in line with the “stress sensitization model” which posits that early caregiving experiences influence children's future vulnerability to later stressful events (McLaughlin et al. 2015; Wade et al. 2019, 2020). Nevertheless, future research is necessary to shed light on the exact mechanisms through which the quality of early maternal care attenuates children's later responses to large‐scale stressors. A direct assessment of children's regulatory capacities and children's sense of security during a lockdown may elucidate important mechanisms underlying the moderating role of early maternal care. Furthermore, in the current study we examined maternal sensitivity, on the basis of extensive research emphasizing sensitivity as a fundamental resilience factor (e.g., Hane and Fox 2006). However, examining other aspects of maternal behavior in infancy, such as playfulness or intrusiveness (e.g., Jung 2011; Moutinho et al. 2023), might be important in broadening our understanding of the impact of infant care on children's future responses to stressors.

In addition, despite previous literature linking maternal stress to compromised maternal behavior (e.g., Crouch and Behl 2001), the present study revealed no significant links between maternal‐reported stress and concurrent maternal sensitivity. Future research is necessary to uncover potential moderators that may play an important role in the translation of maternal stress into maternal behavior. For instance, previous research has suggested that maternal regulatory capacities may be key in conserving optimal caregiver behavior in contexts of stress (e.g., Deater‐Deckard et al. 2009). Likewise, contrary to what we expected, in the present study maternal sensitivity assessed during the lockdown exerted no moderating effect on concurrent child vulnerability to pandemic‐related stressors. Observational measures are rarely used in “disaster” literature (Cobham et al. 2016), and to our knowledge, the present study is the first to report on behavioral observations of maternal care during the pandemic. It is possible that in extreme conditions, such as lockdowns, issues of contextual sensitivity may arise, leading to the compromised validity of our measure. In other words, it may be that maternal sensitivity measured in the context of the videoconference assessment may not have accurately represented the levels of maternal sensitivity actually present in daily life during a lockdown. Supporting this possibility is the fact that no significant association emerged between maternal sensitivity observed during the lockdown and concurrent maternal‐reported stress. Importantly, significant associations did emerge between maternal sensitivity observed in infancy and maternal sensitivity observed during the lockdown. This finding suggests that although maternal behavior observed during the lockdown may have effectively captured stable individual differences in baseline capacities for sensitive caregiving, the effect of these real‐time individual differences may have been washed out in the extreme context of a lockdown. One could hypothesize that even if a parent had the capacity to be sensitive when interacting with a child in a neutral setting, this capacity might not have manifested in the day‐to‐day stressful life routine of a lockdown. Consequently, the basic capacity, which may have been accurately measured, failed to exert protective effects in the face of children's exposure to pandemic‐related stressors. Alternatively, it may be that other aspects of the immediate maternal caregiving behavior were influenced by the lockdown (e.g., reduced time spent with the child, difficulties in maintaining the child's routine), without necessarily diminishing maternal sensitivity. Future research is also necessary to examine additional aspects of real‐time caregiving behavior, which may have played a role in moderating the links between child's DOE and child's concurrent symptoms, such as playfulness (Magnuson and Barnett 2013), harsh parenting (Gershoff et al. 2017; Pinquart 2017), or even the way that parents talked with their child about the stressor (Wilson et al. 2010). Coding of additional aspects of maternal care may tap into more nuanced processes of co‐regulation. In addition, future research should employ additional measurement techniques to assess maternal behavior (e.g., maternal reports regarding behavior and or observations of maternal sensitivity across various contexts during a lockdown). Nevertheless, the significant links that emerged between early maternal sensitive care observed in infancy and later maternal sensitive care observed during the lockdown point to the stability of maternal sensitivity over time and underscore the unique protective effect of early maternal care, which remained significant after controlling for real‐time maternal behavior.

In addition, it is interesting to note that maternal care exerted moderating effects on children's internalizing symptoms, whereas no such effects were found for children's externalizing symptomology. Future research is necessary to elucidate these differential moderating effects on internalizing versus externalizing symptomology. Descriptive data from the present study revealed similar levels of children's internalizing and externalizing symptomology. Additional caregiver factors that were not examined in the present study may indeed have exerted moderating effects on children's externalizing symptoms.

Finally, the present findings should be interpreted in light of several significant methodological limitations. First, the current study's attrition and small sample size must be considered as a clear limitation. Attrition is common in the postpartum period, and the current study's attrition from time of recruitment (during pregnancy) to 4‐months postpartum is comparable with other studies employing postpartum samples (e.g., Fox et al. 2015). Nevertheless, study findings must be interpreted in light of this limitation, and future studies should take additional measures to prevent attrition and employ larger samples. Furthermore, it would be important to use larger samples in the future so that sufficient power is obtained to test one large comprehensive model rather than multiple smaller ones and, as such, avoid potential bias due to multiple comparisons. Second, it should be noted that the inter‐rater reliability assessment of early maternal care was classified as moderate. In addition, the present sample comprised participants from low‐risk, middle‐class, primarily secular Jewish families. Although the sample is reflective of the composition of the population of the region (Israeli Central Bureau of Statistics, 2016), it is not nationally or internationally representative. Homogeneity reduces noise stemming from sociodemographic factors (Bornstein et al. 2013); however, our results may not be generalized. The extent to which other populations are susceptible to these early protective effects remains unknown. Future research should include participants from other socioeconomic backgrounds, as well as clinical populations or samples who experienced an extreme loss of income during the pandemic. In addition, due to a lack of information regarding participants' gender identity, the present study assumed participants had a cisgender identity. Future research should collect information regarding gender identity. An additional limitation of the study stems from the fact that some families may have been differentially affected—for instance, in the event that one or both parents had to work (on the front lines or remotely) – during the lockdown. Variability in parental work obligations during the lockdown may have posed different challenges for families. Although the study was conducted during a nationwide lockdown, during which time all families were subject to the same restrictions and a closure of the educational system, there was still variability that was not measured. Furthermore, assessments of maternal stress, child's mental health symptoms, and child's DOE during the pandemic all relied on maternal reports, which could have been susceptible to reporting bias and may therefore impose limitations stemming from shared method variance. Future research should employ multi‐method data collected from multiple informants, such as teacher reports on child's mental health symptoms, clinical assessments, and behavioral observations. Importantly, given the cross‐sectional nature of the current study, we could not determine the direction of the associations that emerged between maternal stress and child's mental health symptomology. As such, a child's DOE may have had negative effects on a child's mental health symptoms, which may in turn have served to increase maternal stress. Furthermore, the absence of a temporal separation between a child's DOE and a child's subsequent outcome limits our capacity to investigate anything beyond the real‐time impact of the stressor. It is essential to emphasize that our findings, which indicate a correlation between child's DOE and child's outcome, do not inherently imply a causal risk. Rather, this finding underscores the necessity for future studies to investigate the enduring impact of the stressor on a child's long‐term outcomes. Another constraint of the study lies in the fact that we did not account for certain variables from early life that could potentially have influenced our findings—for instance, inclusion of early pre‐ and post‐natal maternal stress levels (e.g., Essex et al. 2002; Talge et al. 2007) and the child's temperamental predisposition at the age of four months (e.g., Kopala‐Sibley et al. 2016). Future studies should include a measurement of these variables. The lack of data on these aspects limits our ability to draw conclusions regarding their potential contribution to the study outcomes. It is also important to note that direct exposure to COVID‐19 has been associated with a medical impact on mental health symptoms (for review, see Shanbehzadeh et al. 2021). Unfortunately, we lacked detailed information regarding individuals' medical status following COVID‐19 exposure. Consequently, we are unable to investigate the influence of the virus itself on the children's emotional states. Finally, the current study did not include fathers, who play a crucial role in family life, particularly during the lockdown when all family members were confined together (e.g., Trumello et al. 2022). Including fathers in future research would contribute to a more comprehensive understanding of family dynamics under stress.

The present findings raise several important questions. Longitudinal follow‐up assessments are necessary to determine the extent to which children's internalizing and/or externalizing symptoms during the lockdown pose a long‐lasting risk for the children's future development. Follow‐up assessments are also necessary to determine the extent to which the early protective effects of infant care are long‐lasting (i.e., into mid‐childhood and later adolescence). Finally, future research is necessary to determine the extent to which timing determines the preventive impact of early care. In other words, is infancy a particularly sensitive time window for the exerting of such protective effects? Or would similar preventive effects emerge in the study of pre‐pandemic caregiving assessed in older children as well? Are the preventive effects of early caregiving particularly salient when provided in infancy, relative to preventive pre‐pandemic care experienced at later ages?

### 6.2 Implications for Developmental Practice and Policy

The present findings are the first to directly demonstrate the principle of an “emotional vaccine” in the face of a large‐scale stressor and to indicate that for preschool‐aged children, significant preventive caregiving experiences may be exerted from as early as infancy. These findings have important policy implications underscoring the importance of investing in the very young and promoting optimal early care (e.g., Heckman 2012). A child's developmental trajectory is the product of an ongoing cumulative interplay between factors of risk and resilience. When a child is faced with an inevitable large‐scale risk factor in the form a global pandemic—and its associated detrimental impact on the child's caregivers—early sensitive caregiving experienced several years prior to the pandemic might serve as the “emotional vaccine” that decreases a young child's vulnerability just enough to make the critical difference. Taken together, the findings emphasize the need to provide caregivers with the necessary support and means to reduce parental stress in the context of large‐scale stressors and warrant the promotion of psychoeducation, preventive efforts, and training of infant caretakers to ensure optimal sensitive caregiving environments in early infancy.

## Conflicts of Interest

The authors declare no conflicts of interest.

## Data Availability

The data and code necessary to reproduce the analyses presented here are publicly accessible, as are the materials necessary to attempt to replicate the findings. Data, code, and materials are available from the corresponding author upon reasonable request. Analyses were not pre‐registered.

## References

[cdev14250-bib-0001] Abidin, R. R. 2012. Parenting Stress Index–Fourth Edition (PSI‐4).

[cdev14250-bib-0002] Abraham, E. , O. Zagoory‐Sharon , and R. Feldman . 2021. “Early Maternal and Paternal Caregiving Moderates the Links Between Preschoolers' Reactivity and Regulation and Maturation of the HPA‐Immune Axis.” Developmental Psychobiology 63, no. 5: 1482–1498. 33432595 10.1002/dev.22089

[cdev14250-bib-0092] Achenbach, T. M . 1991. Manual for the child behaviour checklist/4–18 and 1991 profile. Burlington: University of Vermont Department of Psychiatry.

[cdev14250-bib-0003] Achenbach, T. M. , and A. R. Leslie . 2000. Manual for the ASEBA Preschool Forms and Profiles. Vol. 30. University of Vermont, Research center for children, youth, & families.

[cdev14250-bib-0004] Achterberg, M. , S. Dobbelaar , O. D. Boer , and E. A. Crone . 2021. “Perceived Stress as Mediator for Longitudinal Effects of the COVID‐19 Lockdown on Wellbeing of Parents and Children.” Scientific Reports 11, no. 1: 1–14. 33536464 10.1038/s41598-021-81720-8 PMC7859207

[cdev14250-bib-0005] Adams, E. L. , D. Smith , L. J. Caccavale , and M. K. Bean . 2021. “Parents Are Stressed! Patterns of Parent Stress Across COVID‐19.” Frontiers in Psychiatry 12: 300. 10.3389/FPSYT.2021.626456. PMC8060456 33897489

[cdev14250-bib-0006] Ainsworth, M. D. , S. M. Bell , and D. J. Stayton . 1974. “Infant‐Mother Attachment and Social Development: Socialization as a Product of Reciprocal Responsiveness to Signals.” In The Integration of a Child Into a Social World, edited by M. P. M. Richards , vol. 1, 99–135. Cambridge University Press.

[cdev14250-bib-0007] Albers, E. M. , J. Marianne Riksen‐Walraven , F. C. G. J. Sweep , and C. de Weerth . 2008. “Maternal Behavior Predicts Infant Cortisol Recovery From a Mild Everyday Stressor.” Journal of Child Psychology and Psychiatry 49, no. 1: 97–103. 10.1111/J.1469-7610.2007.01818.X. 18181883

[cdev14250-bib-0008] Asok, A. , K. Bernard , T. L. Roth , J. B. Rosen , and M. Dozier . 2013. “Parental Responsiveness Moderates the Association Between Early‐Life Stress and Reduced Telomere Length.” Development and Psychopathology 25, no. 3: 577–585. 10.1017/S0954579413000011. 23527512 PMC4312590

[cdev14250-bib-0009] Barrett, P. 2007. “Structural Equation Modelling: Adjudging Model Fit.” Personality and Individual Differences 42, no. 5: 815–824. 10.1016/J.PAID.2006.09.018.

[cdev14250-bib-0010] Belsky, J. , and R. M. P. Fearon . 2016. “Precursors of Attachment Security.” In Handbook of Attachment: Theory, Research, and Clinical Applications, edited by J. Cassidy and P. R. Shaver , 291–313. Guilford Press.

[cdev14250-bib-0011] Bernier, A. , S. M. Carlson , and N. Whipple . 2010. “From External Regulation to Self‐Regulation: Early Parenting Precursors of Young Children's Executive Functioning.” Child Development 81, no. 1: 326–339. 10.1111/J.1467-8624.2009.01397.X. 20331670

[cdev14250-bib-0012] Bokszczanin, A. 2008. “Parental Support, Family Conflict, and Overprotectiveness: Predicting PTSD Symptom Levels of Adolescents 28 Months After a Natural Disaster.” Anxiety, Stress, and Coping 21, no. 4: 325–335. 10.1080/10615800801950584. 18686053

[cdev14250-bib-0013] Bornstein, M. H. , J. Jager , and D. L. Putnick . 2013. “Sampling in Developmental Science: Situations, Shortcomings, Solutions, and Standards.” Developmental Review 33, no. 4: 357–370. 10.1016/j.dr.2013.08.003. 25580049 PMC4286359

[cdev14250-bib-0014] Bowlby, J. 1969. Attachment and Loss: Vol. I Attachment. 2nd ed. Basic Books.

[cdev14250-bib-0015] Bowlby, J. 2008. A Secure Base: Parent‐Child Attachment and Healthy Human Development. Basic books.

[cdev14250-bib-0016] Brown, S. M. , J. R. Doom , S. Lechuga‐Peña , S. E. Watamura , and T. Koppels . 2020a. “Stress and Parenting During the Global COVID‐19 Pandemic.” Child Abuse & Neglect 110: 104699. 10.1016/j.chiabu.2020.104699. 32859394 PMC7440155

[cdev14250-bib-0017] Brown, S. M. , L. J. Schlueter , E. Hurwich‐Reiss , J. Dmitrieva , E. Miles , and S. E. Watamura . 2020b. “Parental Buffering in the Context of Poverty: Positive Parenting Behaviors Differentiate Young Children's Stress Reactivity Profiles.” Development and Psychopathology 32, no. 5: 1778–1787. 10.1017/S0954579420001224. 33427174 PMC9118882

[cdev14250-bib-0018] Calkins, S. D. , and A. Hill . 2007. “Caregiver Influences on Emerging Emotion Regulation.” In Handbook of Emotion Regulation, edited by J. J. Gross , 229–248. Guilford Press.

[cdev14250-bib-0019] Chemtob, C. M. , Y. Nomura , K. Rajendran , R. Yehuda , D. Schwartz , and R. Abramovitz . 2010. “Impact of Maternal Posttraumatic Stress Disorder and Depression Following Exposure to the September 11 Attacks on Preschool Children's Behavior.” Child Development 81, no. 4: 1129–1141. 10.1111/j.1467-8624.2010.01458.x. 20636686 PMC3124807

[cdev14250-bib-0020] Chen, X. Y. , J. Chen , X. Shi , et al. 2020. “Trajectories of Maternal Symptoms of Posttraumatic Stress Disorder Predict Long‐Term Mental Health of Children Following the Wenchuan Earthquake in China: A 10‐Year Follow‐Up Study.” Journal of Affective Disorders 266: 201–206. 10.1016/J.JAD.2020.01.084. 32056877

[cdev14250-bib-0021] Chung, G. , P. Lanier , and P. Y. J. Wong . 2020. “Mediating Effects of Parental Stress on Harsh Parenting and Parent‐Child Relationship During Coronavirus (COVID‐19) Pandemic in Singapore.” Journal of Family Violence 37, no. 5: 1–12. 10.1007/s10896-020-00200-1. PMC7467635 32895601

[cdev14250-bib-0022] Chung, G. , J. L. Tilley , N. Netto , A. Chan , and P. Lanier . 2024. “Parenting Stress and Its Impact on Parental and Child Functioning During the COVID‐19 Pandemic: A Meta‐Analytical Review.” International Journal of Stress Management 31: 238–251. 10.1037/STR0000329.

[cdev14250-bib-0023] Cobham, V. E. , B. McDermott , D. Haslam , and M. R. Sanders . 2016. “The Role of Parents, Parenting and the Family Environment in Children's Post‐Disaster Mental Health.” Current Psychiatry Reports 18, no. 6: 1–9. 10.1007/s11920-016-0691-4. 27086314

[cdev14250-bib-0024] Cohodes, E. M. , S. McCauley , and D. G. Gee . 2021. “Parental Buffering of Stress in the Time of COVID‐19: Family‐Level Factors May Moderate the Association Between Pandemic‐Related Stress and Youth Symptomatology.” Research on Child and Adolescent Psychopathology 49, no. 7: 935–948. 10.1007/s10802-020-00732-6. 33591457 PMC7885749

[cdev14250-bib-0025] Comer, J. S. , J. M. Furr , R. S. Beidas , C. L. Weiner , and P. C. Kendall . 2008. “Children and Terrorism‐Related News: Training Parents in Coping and Media Literacy.” Journal of Consulting and Clinical Psychology 76, no. 4: 568–578. 10.1037/0022-006X.76.4.568. 18665686 PMC2728460

[cdev14250-bib-0026] Costa, N. M. , C. F. Weems , and A. A. Pina . 2009. “Hurricane Katrina and Youth Anxiety: The Role of Perceived Attachment Beliefs and Parenting Behaviors.” Journal of Anxiety Disorders 23, no. 7: 935–941. 10.1016/J.JANXDIS.2009.06.002. 19577899

[cdev14250-bib-0027] Crockenberg, S. C. , and E. M. Leerkes . 2004. “Infant and Maternal Behaviors Regulate Infant Reactivity to Novelty at 6 Months.” Developmental Psychology 40, no. 6: 1123–1132. 15535761 10.1037/0012-1649.40.6.1123

[cdev14250-bib-0028] Crouch, J. L. , and L. E. Behl . 2001. “Relationships Among Parental Beliefs in Corporal Punishment, Reported Stress, and Physical Child Abuse Potential.” Child Abuse & Neglect 25, no. 3: 413–419. 10.1016/S0145-2134(00)00256-8. 11414399

[cdev14250-bib-0029] Deater‐Deckard, K. , M. v. Pylas , and S. A. Petrill . 1997. The Parent‐Child Interaction System (PARCHISY).

[cdev14250-bib-0030] Deater‐Deckard, K. , M. D. Sewell , S. A. Petrill , and L. A. Thompson . 2009. “Maternal Working Memory and Reactive Negativity in Parenting.” Psychological Science 21, no. 1: 75–79. 10.1177/0956797609354073. 20424026 PMC2861800

[cdev14250-bib-0031] Dubow, E. F. , L. R. Huesmann , P. Boxer , et al. 2012. “Exposure to Political Conflict and Violence and Posttraumatic Stress in Middle East Youth: Protective Factors.” Journal of Clinical Child & Adolescent Psychology 41, no. 4: 402–416. 10.1080/15374416.2012.684274. 22594697 PMC3387283

[cdev14250-bib-0032] Dyb, G. , T. K. Jensen , and E. Nygaard . 2011. “Children's and Parents' Posttraumatic Stress Reactions After the 2004 Tsunami.” Clinical Child Psychology and Psychiatry 16, no. 4: 621–634. 10.1177/1359104510391048. 21565871

[cdev14250-bib-0033] Essex, M. J. , M. H. Klein , E. Cho , and N. H. Kalin . 2002. “Maternal Stress Beginning in Infancy May Sensitize Children to Later Stress Exposure: Effects on Cortisol and Behavior.” Biological Psychiatry 52, no. 8: 776–784. 10.1016/S0006-3223(02)01553-6. 12372649

[cdev14250-bib-0034] Feldman, R. 1998. Coding Interactive Behavior (CIB) Manual. Unpublished manuscript.

[cdev14250-bib-0035] Feldman, R. , A. Granat , C. Pariente , H. Kanety , J. Kuint , and E. Gilboa‐Schechtman . 2009. “Maternal Depression and Anxiety Across the Postpartum Year and Infant Social Engagement, Fear Regulation, and Stress Reactivity.” Journal of the American Academy of Child and Adolescent Psychiatry 48, no. 9: 919–927. 10.1097/CHI.0b013e3181b21651. 19625979

[cdev14250-bib-0036] Feldman, R. , M. Singer , and O. Zagoory . 2010. “Touch Attenuates Infants' Physiological Reactivity to Stress.” Developmental Science 13, no. 2: 271–278. 10.1111/j.1467-7687.2009.00890.x. 20136923

[cdev14250-bib-0037] Flouri, E. , E. Midouhas , and H. Joshi . 2014. “Family Poverty and Trajectories of Children's Emotional and Behavioural Problems: The Moderating Roles of Self‐Regulation and Verbal Cognitive Ability.” Journal of Abnormal Child Psychology 42, no. 6: 1043–1056. 10.1007/s10802-013-9848-3. 24473936

[cdev14250-bib-0038] Fox, N. A. , N. Snidman , S. A. Haas , K. A. Degnan , and J. Kagan . 2015. “The Relations Between Reactivity at 4 Months and Behavioral Inhibition in the Second Year: Replication Across Three Independent Samples.” Infancy 20, no. 1: 98–114. 10.1111/infa.12063. 25574156 PMC4283938

[cdev14250-bib-0039] Garnett, M. , K. Bernard , J. Hoye , L. Zajac , and M. Dozier . 2020. “Parental Sensitivity Mediates the Sustained Effect of Attachment and Biobehavioral Catch‐Up on Cortisol in Middle Childhood: A Randomized Clinical Trial.” Psychoneuroendocrinology 121: 104809. 10.1016/j.psyneuen.2020.104809. 32781397 PMC7733705

[cdev14250-bib-0040] Gershoff, E. T. , K. M. P. Sattler , and A. Ansari . 2017. “Strengthening Causal Estimates for Links Between Spanking and Children's Externalizing Behavior Problems.” Psychological Science 29: 110–120. 10.1177/0956797617729816. 29106806 PMC5771997

[cdev14250-bib-0041] Gil‐Rivas, V. , R. P. Kilmer , A. W. Hypes , and K. A. Roof . 2010. “The Caregiver‐Child Relationship and Children's Adjustment Following Hurricane Katrina.” In Helping Families and Communities Recover From Disaster: Lessons Learned From Hurricane Katrina and its Aftermath, 55–76. American Psychological Association. 10.1037/12054-002.

[cdev14250-bib-0043] Gunnar, M. R. 2006. “Social Regulation of Stress in Early Child Development.” In Blackwell Handbook of Early Childhood Development, 106–125. Blackwell Publishing.

[cdev14250-bib-0044] Hafstad, G. S. , V. Gil‐Rivas , R. P. Kilmer , and S. Raeder . 2010. “Parental Adjustment, Family Functioning, and Posttraumatic Growth Among Norwegian Children and Adolescents Following a Natural Disaster.” American Journal of Orthopsychiatry 80, no. 2: 248–257. 10.1111/j.1939-0025.2010.01028.x. 20553518

[cdev14250-bib-0045] Hane, A. A. , and N. A. Fox . 2006. “Ordinary Variations in Maternal Caregiving Influence Human Infants' Stress Reactivity.” Psychological Science 17, no. 6: 550–556. 10.1111/j.1467-9280.2006.01742.x. 16771807

[cdev14250-bib-0046] Heckman, J. J. 2012. Invest in Early Childhood Development: Reduce Deficits, Strengthen the Economy, 1–2. Heckman Equation.

[cdev14250-bib-0047] Hu, L. T. , and P. M. Bentler . 2009. “Cutoff Criteria for Fit Indexes in Covariance Structure Analysis: Conventional Criteria Versus New Alternatives.” Structural Equation Modeling: A Multidisciplinary Journal 6, no. 1: 1–55. 10.1080/10705519909540118.

[cdev14250-bib-0048] Jung, J. 2011. “Caregivers' Playfulness and Infants' Emotional Stress During Transitional Time.” Early Child Development and Care 181, no. 10: 1397–1407. 10.1080/03004430.2010.532873.

[cdev14250-bib-0049] Kelley, M. L. , S. Self‐Brown , B. Le , J. V. Bosson , B. C. Hernandez , and A. T. Gordon . 2010. “Predicting Posttraumatic Stress Symptoms in Children Following Hurricane Katrina: A Prospective Analysis of the Effect of Parental Distress and Parenting Practices.” Journal of Traumatic Stress 23, no. 5: 582–590. 10.1002/jts.20573. 20925099 PMC4231140

[cdev14250-bib-0050] Kessel, E. M. , B. D. Nelson , M. Finsaas , et al. 2019. “Parenting Style Moderates the Effects of Exposure to Natural Disaster‐Related Stress on the Neural Development of Reactivity to Threat and Reward in Children.” Development and Psychopathology 31, no. 4: 1589–1598. 10.1017/S0954579418001347. 30724155 PMC6684878

[cdev14250-bib-0051] Kiliç, E. Z. , H. D. Özgüven , and I. Sayil . 2003. “The Psychological Effects of Parental Mental Health on Children Experiencing Disaster: The Experience of Bolu Earthquake in Turkey.” Family Process 42, no. 4: 485–495. 10.1111/j.1545-5300.2003.00485.x. 14979220

[cdev14250-bib-0052] Kopala‐Sibley, D. C. , A. P. Danzig , R. Kotov , et al. 2016. “Negative Emotionality and Its Facets Moderate the Effects of Exposure to Hurricane Sandy on Children's Postdisaster Depression and Anxiety Symptoms.” Journal of Abnormal Psychology 125, no. 4: 471–481. 10.1037/ABN0000152. 27030993 PMC4850107

[cdev14250-bib-0053] Lionetti, F. , M. Spinelli , U. Moscardino , et al. 2022. “The Interplay Between Parenting and Environmental Sensitivity in the Prediction of Children's Externalizing and Internalizing Behaviors During COVID‐19.” Development and Psychopathology 35, no. 3: 1–14. 10.1017/S0954579421001309. 35256026

[cdev14250-bib-0054] Little, R. J. A. 1988. “A Test of Missing Completely at Random for Multivariate Data With Missing Values.” Journal of the American Statistical Association 83, no. 404: 1198–1202. 10.1080/01621459.1988.10478722.

[cdev14250-bib-0055] Ma, L. , M. Mazidi , K. Li , et al. 2021. “Prevalence of Mental Health Problems Among Children and Adolescents During the COVID‐19 Pandemic: A Systematic Review and Meta‐Analysis.” Journal of Affective Disorders 293: 78–89. 10.1016/j.jad.2021.06.021. 34174475 PMC9711885

[cdev14250-bib-0056] Magnuson, C. D. , and L. A. Barnett . 2013. “The Playful Advantage: How Playfulness Enhances Coping With Stress.” Leisure Sciences 35, no. 2: 129–144. 10.1080/01490400.2013.761905.

[cdev14250-bib-0057] Masten, A. S. , and A. J. Narayan . 2012. “Child Development in the Context of Disaster, War, and Terrorism: Pathways of Risk and Resilience.” Annual Review of Psychology 63: 227–257. 10.1146/annurev-psych-120710-100356. PMC5858878 21943168

[cdev14250-bib-0058] McLaughlin, K. A. , M. A. Sheridan , F. Tibu , N. A. Fox , C. H. Zeanah , and C. A. Nelson . 2015. “Causal Effects of the Early Caregiving Environment on Development of Stress Response Systems in Children.” Proceedings of the National Academy of Sciences 112, no. 18: 5637–5642. 10.1073/pnas.1423363112. PMC4426436 25902515

[cdev14250-bib-0042] Mesman, J. , and R. A. G. Emmen 2013. “Mary Ainsworth‘s Legacy: A Systematic Review of Observational Instruments Measuring Parental Sensitivity”. Attachment & Human Development 15: 485–506. 10.1080/14616734.2013.820900. 43–64. 24299131

[cdev14250-bib-0059] Morales, S. , and N. A. Fox . 2019. “A Neuroscience Perspective on Emotional Development.” In Handbook of Emotional Development, 57–81. Springer International Publishing.

[cdev14250-bib-0060] Morris, A. S. , M. M. Criss , J. S. Silk , and B. J. Houltberg . 2017. “The Impact of Parenting on Emotion Regulation During Childhood and Adolescence.” Child Development Perspectives 11, no. 4: 233–238. 10.1111/cdep.12238.

[cdev14250-bib-0061] Moutinho, V. , J. Baptista , A. R. Mesquita , et al. 2023. “Cortisol Reactivity and Negative Affect Among Preterm Infants at 12 Months During a Mother‐Infant Interaction Task.” Infant Behavior and Development 70: 101784. 10.1016/J.INFBEH.2022.101784. 36401957

[cdev14250-bib-0062] Nearchou, F. , E. Hennessy , C. Flinn , R. Niland , and S. S. Subramaniam . 2020. “Exploring the Impact of COVID‐19 on Mental Health Outcomes in Children and Adolescents: A Systematic Review.” International Journal of Environmental Research and Public Health 17, no. 22: 1–19. 10.3390/ijerph17228479. PMC7698263 33207689

[cdev14250-bib-0063] Nelson, C. , N. A. Fox , and C. Zeanah . 2014. Romania's Abandoned Children: Deprivation, Brain Development, and the Struggle for Recovery. Harvard University Press.

[cdev14250-bib-0064] Panchal, U. , G. de Salazar Pablo , M. Franco , et al. 2021. “The Impact of COVID‐19 Lockdown on Child and Adolescent Mental Health: Systematic Review.” European Child & Adolescent Psychiatry 1: 1–27. 10.1007/s00787-021-01856-w. PMC8371430 34406494

[cdev14250-bib-0065] Panda, P. K. , J. Gupta , S. R. Chowdhury , et al. 2021. “Psychological and Behavioral Impact of Lockdown and Quarantine Measures for COVID‐19 Pandemic on Children, Adolescents and Caregivers: A Systematic Review and Meta‐Analysis.” Journal of Tropical Pediatrics 67: no. 1: 1–13. 10.1093/tropej/fmaa122. PMC7798512 33367907

[cdev14250-bib-0066] Pat‐Horenczyk, R. , S. Cohen , Y. Ziv , et al. 2015. “Emotion Regulation in Mothers and Young Children Faced With Trauma.” Infant Mental Health Journal 36, no. 3: 337–348. 10.1002/imhj.21515. 25941026

[cdev14250-bib-0067] Pinquart, M. 2017. “Associations of Parenting Dimensions and Styles With Externalizing Problems of Children and Adolescents: An Updated Meta‐Analysis.” Developmental Psychology 53, no. 5: 873–932. 10.1037/DEV0000295. 28459276

[cdev14250-bib-0068] Racine, N. , R. Eirich , J. Cooke , et al. 2022. “When the Bough Breaks: A Systematic Review and Meta‐Analysis of Mental Health Symptoms in Mothers of Young Children During the COVID‐19 Pandemic.” Infant Mental Health Journal 43, no. 1: 36–54. 10.1002/imhj.21959. 34962649 PMC9015533

[cdev14250-bib-0069] Roberts, M. H. , R. R. Klatzkin , and B. Mechlin . 2015. “Social Support Attenuates Physiological Stress Responses and Experimental Pain Sensitivity to Cold Pressor Pain.” Annals of Behavioral Medicine 49, no. 4: 557–569. 10.1007/s12160-015-9686-3. 25623896

[cdev14250-bib-0070] Rosseel, Y. 2012. “Lavaan: An R Package for Structural Equation Modeling.” Journal of Statistical Software 48: 1–36. 10.18637/jss.v048.i02.

[cdev14250-bib-0071] Russell, B. S. , M. Hutchison , R. Tambling , A. J. Tomkunas , and A. L. Horton . 2020. “Initial Challenges of Caregiving During COVID‐19: Caregiver Burden, Mental Health, and the Parent–Child Relationship.” Child Psychiatry and Human Development 51, no. 5: 671–682. 10.1007/s10578-020-01037-x. 32749568 PMC7398861

[cdev14250-bib-0072] Scheeringa, M. S. , C. H. Zeanah , L. Myers , and F. Putnam . 2004. “Heart Period and Variability Findings in Preschool Children With Posttraumatic Stress Symptoms.” Biological Psychiatry 55, no. 7: 685–691. 10.1016/J.BIOPSYCH.2004.01.006. 15065300

[cdev14250-bib-0073] Schore, A. N. 2001. “Effects of a Secure Attachment Relationship on Right Brain Development, Affect Regulation, and Infant Mental Health.” Infant Mental Health Journal 22, no. 1–2: 7–66. 10.1002/1097-0355(200101/04)22:1<7::AID-IMHJ2>3.0.CO;2-N.

[cdev14250-bib-0074] Schore, J. R. , and A. N. Schore . 2008. “Modern Attachment Theory: The Central Role of Affect Regulation in Development and Treatment.” Clinical Social Work Journal 36, no. 1: 9–20. 10.1007/s10615-007-0111-7.

[cdev14250-bib-0075] Shanbehzadeh, S. , M. Tavahomi , N. Zanjari , I. Ebrahimi‐Takamjani , and S. Amiri‐arimi . 2021. “Physical and Mental Health Complications Post‐COVID‐19: Scoping Review.” Journal of Psychosomatic Research 147: 110525. 10.1016/j.jpsychores.2021.110525. 34051516 PMC8133797

[cdev14250-bib-0076] Shorer, M. , and L. Leibovich . 2020. “Young Children's Emotional Stress Reactions During the COVID‐19 Outbreak and Their Associations With Parental Emotion Regulation and Parental Playfulness.” Early Child Development and Care 192, no. 6: 1–11. 10.1080/03004430.2020.1806830.

[cdev14250-bib-0077] Smyke, A. T. , C. H. Zeanah , N. A. Fox , C. A. Nelson , and D. Guthrie . 2010. “Placement in Foster Care Enhances Quality of Attachment Among Young Institutionalized Children.” Child Development 81, no. 1: 212–223. 10.1111/J.1467-8624.2009.01390.X. 20331663 PMC4098033

[cdev14250-bib-0078] Spell, A. W. , M. L. Kelley , J. Wang , et al. 2008. “The Moderating Effects of Maternal Psychopathology on Children's Adjustment Post–Hurricane Katrina.” Journal of Clinical Child & Adolescent Psychology 37, no. 3: 553–563. 10.1080/15374410802148210. 18645746

[cdev14250-bib-0079] Spinelli, M. , F. Lionetti , M. Pastore , and M. Fasolo . 2020. “Parents' Stress and Children's Psychological Problems in Families Facing the COVID‐19 Outbreak in Italy.” Frontiers in Psychology 11: 1713. 10.3389/fpsyg.2020.01713. 32719646 PMC7350926

[cdev14250-bib-0080] Stracke, M. , M. Heinzl , A. D. Müller , et al. 2023. “Mental Health Is a Family Affair—Systematic Review and Meta‐Analysis on the Associations Between Mental Health Problems in Parents and Children During the COVID‐19 Pandemic.” International Journal of Environmental Research and Public Health 20, no. 5: 4485. 10.3390/IJERPH20054485/S1. 36901492 PMC10001622

[cdev14250-bib-0081] Talge, N. M. , C. Neal , and V. Glover . 2007. “Antenatal Maternal Stress and Long‐Term Effects on Child Neurodevelopment: How and Why?” Journal of Child Psychology and Psychiatry 48, no. 3–4: 245–261. 10.1111/J.1469-7610.2006.01714.X. 17355398 PMC11016282

[cdev14250-bib-0082] Tang, S. , M. Xiang , T. Cheung , and Y. T. Xiang . 2021. “Mental Health and Its Correlates Among Children and Adolescents During COVID‐19 School Closure: The Importance of Parent‐Child Discussion.” Journal of Affective Disorders 279: 353–360. 10.1016/j.jad.2020.10.016. 33099049 PMC7550131

[cdev14250-bib-0083] Troy, A. S. , and I. B. Mauss . 2011. “Resilience and Mental Health: Challenges Across the Lifespan—Google Books.” In Resilience and Mental Health: Challenges Across the Lifespan, edited by S. M. Southwick , B. T. Litz , D. Charney , and M. J. Friedman , 30–44. Cambridge University Press. https://books‐google‐co‐il.ezprimo1.runi.ac.il/books?hl=en&lr=&id=lY2GRulPCiMC&oi=fnd&pg=PA30&dq=emotion+regulation+support+adaptive+response+to+everyday+stressors+&ots=ntZzyWt3Ar&sig=FM1QefumSAZNWMCtFJM6yo3jpmM&redir_esc=y#v=onepage&q=emotion%20regulation%20support%20adaptive%20response%20to%20everyday%20stressors&f=false.

[cdev14250-bib-0084] Trumello, C. , S. M. Bramanti , L. Lombardi , et al. 2022. “COVID‐19 and Home Confinement: A Study on Fathers, Father–Child Relationships and Child Adjustment.” Child: Care, Health and Development 48, no. 6: 917–923. 10.1111/CCH.12912. 34510515 PMC8653319

[cdev14250-bib-0085] Valiente, C. , R. A. Fabes , N. Eisenberg , and T. L. Spinrad . 2004. “The Relations of Parental Expressivity and Support to Children's Coping With Daily Stress.” Journal of Family Psychology 18, no. 1: 97–106. 10.1037/0893-3200.18.1.97. 14992613

[cdev14250-bib-0086] Wade, M. , M. A. Sheridan , C. H. Zeanah , N. A. Fox , C. A. Nelson , and K. A. McLaughlin . 2020. “Environmental Determinants of Physiological Reactivity to Stress: The Interacting Effects of Early Life Deprivation, Caregiving Quality, and Stressful Life Events.” Development and Psychopathology 32, no. 5: 1732–1742. 10.1017/S0954579420001327. 33427173 PMC7934448

[cdev14250-bib-0087] Wade, M. , C. H. Zeanah , N. A. Fox , F. Tibu , L. E. Ciolan , and C. A. Nelson . 2019. “Stress Sensitization Among Severely Neglected Children and Protection by Social Enrichment.” Nature Communications 10, no. 1: 1–8. 10.1038/s41467-019-13622-3. PMC6920417 31852902

[cdev14250-bib-0088] Wickrama, K. A. S. , and V. Kaspar . 2007. “Family Context of Mental Health Risk in Tsunami‐Exposed Adolescents: Findings From a Pilot Study in Sri Lanka.” Social Science & Medicine 64, no. 3: 713–723. 10.1016/j.socscimed.2006.09.031. 17084953

[cdev14250-bib-0089] Williamson, V. , C. Creswell , P. Fearon , R. M. Hiller , J. Walker , and S. L. Halligan . 2017. “The Role of Parenting Behaviors in Childhood Post‐Traumatic Stress Disorder: A Meta‐Analytic Review.” Clinical Psychology Review 53: 1–13. 10.1016/J.CPR.2017.01.005. 28137661

[cdev14250-bib-0090] Wilson, A. C. , L. J. Lengua , A. N. Meltzoff , and K. A. Smith . 2010. “Parenting and Temperament Prior to September 11, 2001, and Parenting Specific to 9/11 as Predictors of Children's Posttraumatic Stress Symptoms Following 9/11.” Journal of Clinical Child & Adolescent Psychology 39, no. 4: 445–459. 20589557 10.1080/15374416.2010.486317 PMC2897067

[cdev14250-bib-0091] Wolchik, S. A. , K. L. Wilcox , J. Y. Tein , and I. N. Sandler . 2000. “Maternal Acceptance and Consistency of Discipline as Buffers of Divorce Stressors on Children's Psychological Adjustment Problems.” Journal of Abnormal Child Psychology 28, no. 1: 87–102. 10.1023/A:1005178203702. 10772352

